# Vegan and vegetarian males and females have higher orthorexic traits than omnivores, and are motivated in their food choice by factors including ethics and weight control

**DOI:** 10.1177/02601060231187924

**Published:** 2023-07-19

**Authors:** Rebecca Reynolds, Andrea McGowan, Sophie Smith, Patrick Rawstorne

**Affiliations:** 1 School of Population Health, Faculty of Medicine, UNSW, Sydney, NSW, Australia; 2 University of Michigan Medical School, Ann Arbor, MI, USA

**Keywords:** Vegans, vegetarians, motivation, feeding behaviour, feeding and eating disorders, orthorexia nervosa

## Abstract

**Background:**

Evidence associating plant-based diets with the proposed ‘obsessively healthy eating’ eating disorder, orthorexia nervosa, has mostly focused on females. Diet motivations have seldom been assessed.

**Aim:**

To compare orthorexic tendencies between vegans/vegetarians and omnivores of both sexes, and reasons behind food choice with an English-validated Food Choice Questionnaire.

**Methods:**

A cross-sectional survey of 444 males and females were recruited via social media, email, and Amazon MTurk; to investigate eating patterns, orthorexic tendencies using the ORTO-15 questionnaire, and eating motivations using the Food Choice Questionnaire.

**Results:**

Over half of the participants were male (53.4%), younger adults (mean ± SD 37.2 ± 11.2 years), and mostly from the United States (89%). Vegan and vegetarian eating habits were reported by 15.8% of people. Vegans/vegetarians had significantly higher orthorexic tendencies than omnivores, and chose food significantly more often for Weight Control, Ethical Concern, Natural Content, and Mood reasons. People with greater orthorexic tendencies (ORTO-15 score<35) chose food significantly more often for Weight Control and Ethical Concern reasons than those with less orthorexic tendencies (ORTO-15 score 35+).

**Conclusion:**

This study's results are in line with the majority of the evidence that shows an association between vegan/vegetarian diets and orthorexic tendencies, but strengthens the evidence base by including more male participants. Additionally, this is the first study to use an English-validated motivation-based questionnaire that explored diet motivators in vegans/vegetarians compared to omnivories, and in those with orthorexic tendencies vs. those without orthorexic tendencies.

## Introduction

Orthorexia nervosa (ON) was originally proposed as an eating disorder in 1997 by Steven Bratman and involves a fixation on dietary healthiness (Bratman, 1997). In recent years, there has been an increase in research into the condition; but there has been debate on its definition, a psychometric instrument robust enough to assess it, and diagnostic criteria (Cena et al., 2019). One survey of health professionals in Australia and New Zealand reported that 71% of them believed that ON should be a distinct, clinically recognised eating disorder (Reynolds & McMahon, 2020), highlighting the importance of continued research into it.

Very recently, a panel of 47 eating disorder researchers and multidisciplinary treatment specialists from around the globe contributed to a consensus document on ON, proposing 27 statements on the definition and diagnosis of the disorder. Excerpts from these statements highlight that in ON, there is ‘a strong preoccupation with one's eating behaviour and with self-imposed rigid and inflexible rules’ and that the ‘definition of ‘healthful eating’ or ‘pure eating’ indicates a dietary theory or set of beliefs whose specific details may vary’ (Donini et al., 2022). The panel also agreed that ON is a distinct eating disorder associated with imparied health status and reduced wellbeing, and that appearance and weight concerns are not central to ON, unlike in the eating disorder, anorexia nervosa (Donini et al., 2022).

There are several types of vegetarian-style diets, including lacto-ovo-vegetarianism and veganism (Table 1). These dietary patterns may be rising in popularity in Australia, with market research showing that the percentage of Australians whose diet is all or almost all vegetarian increased from 9.7% in 2012 to 11.2% in 2016 (Roy Morgan, 2016). Driving factors for such plant-based eating include health, ethics, and taste (Rosenfeld & Burrow, 2017). Also, dietary meat avoidance has been linked to disordered eating and eating disorders, with one study reporting that females with a history of an eating disorder were more likely to have been vegetarian at some point in their lives compared with females with no eating disorder history (Bardone-Cone et al., 2012). However, a 2022 systematic review of 48 studies investigating the association between vegetarianism, veganism, and disordered eating noted that there was no consensus on whether meat avoidance was linked to more disordered eating; but that most of the 18 studies that looked at ON specifically showed positive associations with vegetarianism and veganism – mainly in females (McLean et al., 2022). Three of these 18 studies also investigated reasons behind food choice, with mixed results (Barthels et al., 2020, Hessler-Kaufmann et al., 2020, Parra-Fernández et al., 2020), and only one study using a validated motivation-based questionnaire (in Spanish) (Parra-Fernández et al., 2020). Barthels et al. (2020) reported that orthorexic tendencies were associated with higher importance placed on the diet motivators of health, aesthetics (e.g., improved appearance of skin), and healing (e.g., of an illness). Conversely, orthorexic tendencies were associated with lower importance placed on the diet motivators of mood, health, and weight control in the Parra-Fernández et al. (2020) study. Additionally, Hessler-Kaufmann et al. (2020) reported that vegetarians had significantly higher orthorexic traits and ecological and ethical eating motives compared to semi-vegetarians and omnivores.

**Table 1.** Seven vegan/vegetarian dietary pattern sub-groups.^1^

Dietary vegan	Never consume meat, dairy or eggs; but use non-vegan clothing and beauty products
Lifestyle and dietary vegan	Never consume meat, dairy, eggs or honey; and avoid usage of beauty products and clothing that exploit or harm animals
Lacto-ovo-vegetarian	Never consume meat, fish or fowl (e.g., chicken); but consume eggs and dairy
Pescatarian	Never consume meat or fowl (e.g., chicken); but consume fish, eggs and dairy
Lacto-vegetarian	Never consume eggs, meat, fish or fowl (e.g., chicken); but consume dairy
Ovo-vegetarian	Never consume dairy, meat, fish or fowl (e.g., chicken); but consume eggs
Flexitarian/semi-vegetarian	Consume meat, fish or fowl (e.g., chicken) more than 1 time per month, but less than 1 time per week; and eggs/dairy at any frequency^2^

^1^Classifications based on those provided by the Dietitians Australia (2017) and Hennigan (2015). ^2^Frequency obtained from Derbyshire (2016).

The aims of the present cross-sectional survey were to compare orthorexic tendencies between a sample of vegans/vegetarians and omnivores of both sexes and reasons behind food choice with an English-validated Food Choice Questionnaire (FCQ). We hypothesised that vegans/vegetarians would have higher orthorexic traits than omnivores, and would choose food more often for ethical reasons. We also hypothesised that those with higher orthorexic traits would choose food more often for health reasons. This is the first study to explore ON, food choice motivations, and plant-based eating habits in an international population of both sexes using a validated English food choice tool.

## Methods

### Study design

This online survey study was cross-sectional in design and received ethics approval from UNSW Sydney’s Human Research Ethics Committee, approval number HC17775. The survey was hosted by Qualtrics.com and data were collected between November 2017 and October 2018.

### Participant inclusion and recruitment

The inclusion criteria for participants were: 16 years of age or older and first language being English. Participants were recruited via social media, email, and Amazon Mechanical Turk (MTurk: an online platform that enables researchers to ‘crowdsource’ participants to fill in online surveys, for example, in return for reimbursement).

Two Facebook pages and two Instagram accounts were set up for the study, one site on each social media platform targeting vegetarian eaters, and the other omnivores. In the descriptions beneath the picture posts, hashtags and captions were used to try to reach participants, e.g., ‘*Support the vegan and vegetarian community AND go in the running for a voucher. Answer this quick survey! #vegan #vegetarian …*’, with subsequent information clearly outlining the study's aims of exploring motivations behind different eating patterns and any links with obsessively healthy eating. On Instagram, participants were also recruited with a more targeted approach, similar to that conducted by Holland and Tiggemann (2017). This included identifying Instagram users that had biographies that seemed to fit our target demographic by following them, liking one of their pictures, and then sending an invitation to participate in the study in a comment on the liked picture. Via Facebook, we also posted pictures and accompanying information on the walls of Facebook pages that seemed relevant to our study, e.g., the Australian Vegetarian Society. The administrator of such pages had to approve our post before it would be shown to the page's audience.

Emails were sent to the organisations that seemed to be relevant to the study by researchers AM and RR (such as the national charity, Vegan Australia) to ask the organisation to send a recruitment email to their contact databases, or to provide information about the study in a newsletter or on a social media page.

We struggled to get enough participants to complete the survey through the targeted recruitment means of social media and email (from November 2017), hence we expanded recruitment to include MTurk (with tasks posted in March 2018 and October 2018) by posting tasks that stated ‘Give us your opinion about what you eat – are you a vegetarian or vegan; or are you an ‘omnivore’ and eat both plant and animal foods?’, with the following keywords listed: Survey, diet, eating, nutrition, health, vegetarian, vegan, omnivore, food.

Participants who completed the survey and who lived in Australia were eligible to win an AUD$50 voucher for an online store that delivered only to Australia (The Cruelty Free Shop) in a prize draw that aimed to reimburse participants for their time. Amazon MTurk participants were also paid USD$1 to complete the survey.

### Online survey

Informed consent was obtained from all individual participants, and the Participant Information Statement and Consent Form stated the following: ‘We don’t expect this questionnaire to cause any harm or discomfort, however, if you experience feelings of distress as a result of participation in this study (e.g., related to disordered eating habits), you can let the research team know and they will provide you with assistance. Alternatively, you can contact the organisations below for support’ – with mental health support details listed beneath in Australia, America, and beyond.

The online Qualtrics survey assessed the following.

### General

Demographics (age, sex, place of residence, highest level of education, and ethnicity), height and weight (body mass index (BMI) was calculated as weight (kg)/height (m)^2^), and current or past diagnosis of a Diagnostic and Statistical Manual of Mental Disorders-5-defined eating disorder (American Psychiatric Association, 2013). We asked about current or past diagnosis of an eating disorder to see if there was an association between it and diet pattern, as per previous research (Bardone-Cone et al., 2012).

### Diet

Plant- or animal-centric eating categories and frequencies were based on information provided by the Dietitians Australia (2017), Hennigan (2015), and Derbyshire (2016). Participants were first asked to classify themselves into one of two groups, either vegans/vegetarians or omnivores, with subsequent questions asking for further details regarding each way of eating to create sub-groups. Table 1 illustrates the seven vegan/vegetarian sub-groups. In comparison, there were only three omnivore sub-categories – *Animal-heavy omnivore*: Relying more on animal foods/drinks than plant foods/drinks for nutrition (i.e., consume animal foods or drinks 5 + times per day); *Animal-moderate omnivore*: Relying equally on animal and plant foods/drinks for nutrition (i.e., consume animal foods or drinks 3–4 times per day); *Plant-heavy omnivore*: relying more on plant foods/drinks than animal foods/drinks for nutrition (i.e., consume animal foods or drinks ≤ 2 times per day).

### Orthorexic tendencies

The ORTO-15 questionnaire (Donini et al., 2005) was administered to indicate orthorexic tendencies and estimated prevalence of ON. Answers to the 15 items were on a four-point scale of ‘1 never’ to ‘4 always’ (an item example is ‘Do you think that eating healthy food changes your lifestyle (frequency of eating out, friends, …)?’). Scores were summed and used to estimate a point prevalence of the proposed condition, with the more conservative cut-off value of < 35 being used in the current study to indicate ON. The ORTO-15 showed an acceptable level of internal consistency reliability in our study (α=0.79), comparable with other studies that have used the instrument (Varga et al., 2014).

### Motivators behind food choice

The FCQ assessed eating motivations (Steptoe et al., 1995). In response to the sentence, ‘*It is important to me that the food I eat on a typical day …’*, there are 36 statements with answer ratings on a four-point scale, from ‘1 not at all important’ to ‘4 very important’. Therefore, higher scores indicate greater motivation importance. The statements are grouped into nine motivational dimensions, each of three to six items: Health (e.g., ‘*…* keeps me healthy’), Mood (e.g., ‘… helps me to cope with life’), Convenience (e.g., ‘… can be bought in shops close to where I live or work’), Sensory Appeal (e.g., ‘… has a pleasant texture’), Natural Content (e.g., ‘… contains no artificial ingredients’), Price (e.g., ‘… is cheap’), Weight Control (e.g., ‘… helps me control my weight’), familiarity (e.g., ‘… is like the food I ate when I was a child’), and Ethical Concern (e.g., ‘… has the country of origin clearly marked’) (Steptoe et al., 1995). Scores for each dimension are averaged. The FCQ showed moderate to strong levels of internal consistency reliability for each of the nine dimensions: Health α=0.87, Mood α=0.88, Convenience α=0.77, Sensory Appeal α=0.73, Natural Content α=0.89, Price α=0.80, Weight Control α=0.76, Familiarity α=0.77, and Ethical Concern α=0.78.

We added five additional statements in the Ethical Concern dimension, because we wanted to better understand ethical motivator details: ‘… does not involve the harm or exploitation of animals’, ‘… does not involve the killing of animals’, ‘… is more environmentally friendly’, ‘… is fair-trade’, and ‘… is organic’.

We added one extra question that asked which one dimension from the FCQ was the *single most* important in food selection: Health, Mood, Convenience, Sensory Appeal, Natural Content, Price, Weight Control, Familiarity, and Ethical Concern (Ethical Concern was divided into four categories: Ethical Concern for Animal Welfare, Ethical Concern for the Environment, Ethical Concern for Fair Trading, and Organic Produce).

### Statistical approach

#### Hypotheses

The study's hypotheses were specified before the data were collected. We hypothesised that people with plant-focused eating habits would score lower on the ORTO-15, i.e., have higher orthorexic tendencies, consistent with previous findings (Rosenfeld & Burrow, 2017); as well as chose food more often for ethical reasons because plant-based diets are often motivated by personal ethics (Rosenfeld & Burrow, 2017). We also hypothesised that those with higher orthorexic traits would chose food more often for health reasons, as this is a central motivator in the proposed eating disorder (Bratman, 1997).

#### Sample size

To calculate sample size, we used two mean scores obtained with the ORTO-15 questionnaire for younger people (mainly Italian university students) in a study by Dell’Osso et al. (2016) that also assessed diet pattern (including vegetarian/vegan). We chose the young adult demographic because we hypothesised that this population group would be most likely to respond to our survey because younger people are heavier users of social media (Global Web Index, 2022). We used the mean values obtained on the ORTO-15 questionnaire for those who had no dietary restrictions (35.23) vs. vegans/vegetarians (37.37), and the average of the two standard deviations (3.98 + 4.81/2 = 4.40). Using a sampling ratio of 1 and assuming equal variance, we estimated that we would require at least 66 participants in each group, i.e., 132 in total.

#### Data cleaning

There were 541 responses recorded by Qualtrics, and most of these participants were recruited via MTurk because there were 500 survey ‘assignments’ completed there. The following number of survey responses were excluded, with reasons also listed: *n* = 3 under 16 years of age; *n* = 25 first language, not English; unrealistic height, weight, or BMI as per the Australian Household, Income and Labour Dynamics in Australia survey (Wooden et al., 2008): *n* = 42 height < 120 cm or > 210 cm, *n* = 14 weight <40 kg or >200 kg, *n* = 13 BMI <15 or >50. This left a total of 444 completed questionnaires.

#### Statistical analysis

Results were analysed using IBM's SPSS statistical software. Descriptive statistics were calculated, with normalities of distribution checked using Shapiro-Wilk Tests. The following variables were not normally distributed in either the vegan/vegetarian group or the omnivore group: age, BMI, ORTO-15 sum cut-off point of <35, and the following motivational dimensions of the FCQ: Mood, Convenience, Sensory, Natural, Price, Weight, and Familiarity. The following variables were not normally distributed for the omnivore dietary pattern: ORTO-15 sum and the Health and the Ethical domains of the FCQ. Therefore, most statistical analyses were conducted using non-parametric methods.

The primary outcomes were differences in total ORTO-15 score and prevalence of ON using the cut-off total score of <35 between vegan/vegetarian and omnivore groups. The secondary outcomes were average scores in each of the nine motivational dimensions on the FCQ. We *did not* include the extra five questions on Ethical Concern that we added to the FCQ when calculating FCQ score averages for the Ethical Concern domain. Differences between groups were calculated using Pearson's chi-squared χ^2^ tests for categorical variables, or unpaired Mann-Whitney *U* tests for ordinal variables where there were two groups (e.g., vegan/vegetarian vs. omnivore groups), or Kruskal–Wallis H χ^2^ tests where there were three or more groups (e.g., people grouped into the ten dietary sub-groups). Pearson correlation coefficients *r* between selected variables of interest were also explored.

## Results

There were 444 completed surveys. Data are available at this link: https://doi.org/10.6084/m9.figshare.21006490.v2

### Participant characteristics and dietary patterns

#### Characteristics

The age range of participants was 16–74 years, with a mean age of 37.2 ± 11.2 years. The BMI range was 15–50 kg/m^2^, mean 27.1 ± 6.6 kg/m^2^. The remaining participant characteristics are illustrated in Table 2.

**Table 2.** Participant characteristics.

		*n* (%) of all participants (*n* = 444)
Sex	Male	237 (53.4%)
	Female	202 (45.5%)
	Other	3 (0.7%)
	Missing	2 (0.5%)
Place of residence	US	395 (89.0%)
	Other	21 (4.7%)
	India	11 (2.5%)
	Australia	11 (2.5%)
	UK	5 (1.1%)
	Canada	1 (0.2%)
Highest level of education	Bachelor's degree or above	262 (59.0%)
	Diploma or similar	106 (23.9%)
	Finished high school	72 (16.2%)
	Did not go to or finish high school	4 (0.9%)
Race	Caucasian	254 (57.2%)
	Southeast Asian	42 (9.5%)
	Other	60 (13.5%)
	East Asian	33 (7.4%)
	African	19 (4.3%)
	Latino/Hispanic	11 (2.5%)
	South Asian	10 (2.3%)
	Mixed	6 (1.4%)
	Missing	6 (1.4%)
	Middle Eastern	3 (0.7%)
Current or past diagnosis of an eating disorder (participants could choose more than one option)	Yes	**57** (**12.8%)**
	- Pica	- 15 (3.4%)
	- Binge eating disorder	- 14 (3.2%)
	- Anorexia nervosa	- 9 (2.0%)
	- Bulimia nervosa	- 9 (2.0%)
	- Other specified feeding and eating disorder	- 4 (0.9%)
	- Avoidant/restrictive food intake disorder	- 3 (0.7%)
	- Unspecified feeding or eating disorder	- 2 (0.5%)
	- Rumination disorder	- 1 (0.2%)

#### Summary table of results

Please see Table 3 for a summary of the study's main results, which are further detailed in the following results sections.

**Table 3.** Summary of study's main results.

Variable		VV *n*/total *n*	OM *n*/total *n* or ORTO-15 score mean ± SD	Statistical test result and significance	Take-home message
*n* (%)		70/444 (15.8%)	374/444 (84.2%)	-	15% of sample of 444 were VV, 84.2% were OM
Sex	Male	39/70	-	χ^2^(3) = 1.048, *p* = 0.790	NS effect of sex on vv vs. OM diet pattern
	Female	31/70	-		
Current or past diagnosis of an ED	Yes	18/70	-	χ^2^(1) = 12.314, *p* = 0.000	Current or past diagnosis of an ED significantly higher than expected counts of VV compared to OM
	No	52/70	-		
ORTO-15 score		38.9 ± SD 8.1	41.5 ± 6.3	*Z* = −2.387, *p* = 0.017	ORTO-15 score significantly lower in VV vs. OM, i.e., greater ON tendencies
ORTO-score <35		22/70	44/374	χ^2^(1) = 18.016, *p* = 0.000	*Relatively* more participants in VV group scored ORTO-15 < 35 (i.e., greater ON tendencies) compared to OM group (*31.4% VV vs. 11.8% OM)*
Variable		VV FCQ mean ± SD score	OM FCQ mean ± SD score	Statistical test result and significance	-
FCQ Mood		2.63 ± 0.78	2.30 ± 0.75	*Z* = −3.286, *p* = 0.001	VV group placed greater importance on Mood as a food choice motivator than OM group
FCQ Natural Content		2.91 ± 0.78	2.43 ± 0.90	*Z* = −4.193, *p* = 0.000	VV group placed greater importance on Natural Content as a food choice motivator than OM group
FCQ Weight Control		2.59 ± 0.75	2.31 ± 0.79	*Z* = −2.959, *p* = 0.003	VV group placed greater importance on Weight Control as a food choice motivator than OM group
FCQ Ethical Concern		2.52 ± 0.78	1.95 ± 0.79	*Z* = −5.447, *p* = 0.000	VV group placed greater importance on Ethical Concern as a food choice motivator than OM group
FCQ Health	ORTO-15 score <35 YES	2.98 ± 0.63	-	*Z* = −2.151, *p* = 0.032	Participants who scored ORTO-15 < 35 (i.e., greater ON tendencies) placed greater importance on Health as a food choice motivator than those who scored 35 + on the ORTO-15
	ORTO-15 score <35 NO	2.76 ± 0.71	-		
FCQ Weight Control	ORTO-15 score <35 YES	2.81 ± 0.66	-	*Z* = −5.082, *p* = 0.000	Participants who scored ORTO-15 < 35 (i.e., greater ON tendencies) placed greater importance on Weight Control as a food choice motivator than those who scored 35 + on the ORTO-15
	ORTO-15 score <35 NO	2.28 ± 0.78	-		

ED: eating disorder; FCQ: Food Choice Questionnaire (Steptoe et al., 1995); NS: non-significant; SD: standard deviation; OM: omnivore; ON: orthorexia nervosa; ORTO-15: score on ORTO-15 questionnaire (Donini et al., 2005); VV: vegan/vegetarian.

#### Dietary patterns

Table 4 illustrates the dietary patterns of participants, with *n* = 70 (15.8%) of our sample following either vegan/vegetarian eating habits compared to omnivorous eating, *n* = 374 (84.2%). When looking at dietary pattern sub-groups, most of the omnivore group were either animal-heavy (*n* = 76/374, 20.3%) or animal-moderate (*n* = 223/374, 59.6%). In the vegan/vegetarian category, *n* = 34/70 (48.6%) participants stated that their diet pattern was either of the two vegan diet sub-groups, *n* = 28/70 (40.0%) any of the three vegetarian diet sub-groups, *n* = 7/70 (10.0%) the pescatarian sub-group, and *n* = 1/70 (1.4%) the flexitarian/semi-vegetarian sub-group.

**Table 4.** Dietary patterns and ON

Dietary pattern (*n*, % of all dietary patterns)		ORTO-15 total score, mean ± SD	ORTO-15 total score < 35, *n*/total *n* (estimated prevalence ON %)
All participants (444, 100%)		41.1 ± 6.6	66/444 (14.9%)
Omnivores	All of omnivore group (374, 84.2%)	41.5 ± 6.3	44/374 (11.8%)
	Animal-heavy omnivore (76, 17.1%)	41.8 ± 7.1	13/76 (17.1%)
	Animal-moderate omnivore (223, 50.2%)	41.6 ± 6.1	22/223 (9.9%)
	Plant-heavy omnivore (75, 16.9%)	40.8 ± 5.8	9/75 (12.0%)
Vegan/vegetarians	All of vegan/vegetarian group (*n* = 70, 15.8%)	38.9 ± 8.1	22/70 (31.4%)
	Flexitarian/semi-vegetarian (1, 0.2%)	40.0	0/1 (0.0%)
	Pescatarian (7, 1.6%)	37.9 ± 5.0	2/7 (28.6%)
	Lacto-ovo-vegetarian (20, 4.5%)	41 ± 6.6	5/20 (25.0%)
	Lacto-vegetarian (6, 1.4%)	36.5 ± 11.9	2/6 (33.3%)
	Ovo-vegetarian (2, 0.5%)	39.5 ± 4.9	0/2 (0.0%)
	Dietary vegan (21, 4.7%)	37.7 ± 10.5	9/21 (42.9%)
	Lifestyle and dietary vegan (13, 2.9%)	39.2 ± 6.2	4/13 (30.8%)

ON, orthorexia nervosa; ORTO-15, score on ORTO-15 questionnaire (Donini et al., 2005); SD, standard deviation.

#### Influence of selected participant characteristics on vegan/vegetarian vs. omnivore dietary patterns

*Sex:* Did not influence the high-level dietary pattern of vegan/vegetarian vs. omnivore: Pearson chi-square χ^2^(3) = 1.048, *p* = 0.790 (*n* vegan/vegetarian males = 39/237 and females = 31/202). *Current or past diagnosis of an eating disorder:* Pearson chi-square χ^2^(1) = 12.314, *p* = 0.000, with those with a current or past diagnosis of an eating disorder having higher than expected counts of vegan/vegetarian eating habits compared to omnivores (*n* vegan/vegetarians with a current or past diagnosis of an eating disorder = 18/70 vs. omnivores with a current or past diagnosis of an eating disorder = 52/70).

#### The ORTO-15 questionnaire

Table 4 also shows ORTO-15 questionnaire (Donini et al., 2005) scores to indicate orthorexic tendencies, and the estimated prevalence of ON based on the ORTO-15 sum cut-off point of <35. Results ranged from a minimum score of 20.0 to a maximum score of 60.0 in all participants.

A Mann-Whitney *U* test showed a statistically significant difference in ORTO-15 score between the two main dietary patterns of vegan/vegetarian vs. omnivore, *Z* = −2.387, *p* = 0.017 (vegan/vegetarian mean ORTO-15 score = 38.9 ± SD 8.1 vs. omnivore mean ORTO-15 score = 41.5 ± SD 6.3). However, a Kruskal–Wallis H test showed no statistically significant difference in ORTO-15 score between the ten different dietary sub-groups, χ^2^(9) = 7.555, *p* = 0.579.

A Pearson's chi-squared χ^2^ test showed a statistically significant difference in the number of participants who scored <35 in the ORTO-15 questionnaire vs. those who scored 35 + between the two main dietary patterns of vegan/vegetarian vs. omnivore, χ^2^(1) = 18.016, *p* = 0.000 (vegan/vegetarian *n* = 22/70 (31.4%) vs. omnivore *n* = 44/374 (11.8%)). Additionally, a Kruskal–Wallis H test showed significant differences between all ten eating sub-patterns (χ^2 ^= 25.539, *p* = 0.002) in the number of participants who scored <35 on the ORTO-15 vs. those who scored 35+: The highest estimated prevalence of ON was in dietary vegans (*n* = 9/21, 42.9%), followed by lacto-vegetarians (*n* = 2/6, 33.3%), lifestyle and dietary vegans (*n* = 4/13, 30.8%), pescatarians (*n *= 2/7, 28.6%), lacto-ovo-vegetarians (*n* = 5/20, 25%), animal-heavy omnivores (*n* = 13/76, 17.1%), plant-heavy omnivores (*n* = 9/75, 12.0%), animal-moderate omnivores (*n* = 22/223, 9.9%), and flexitarian/semi-vegetarians and ovo-vegetarians (*n* = 0/1, 0%).

Pearson correlations between ORTO-15 score and FCQ Health Concern and Weight Control scores were significant, small to medium, and negative: Health Concern: *r* = −0.242, *p* = 0.000; Weight Control *r* = −0.325, *p* = 0.000.

#### Food choice motivators

Table 5 illustrates the motivating factors behind food choice, as assessed by the FCQ (Steptoe et al., 1995) and using our additional questions that were on ethical concern, for all participants and between main diet groups (omnivore and vegan/vegetarian). Table 6 shows the single most important motivating factor from the nine domains of the FCQ that was chosen by all participants and between main diet groups (omnivore and vegan/vegetarian).

**Table 5.** Motivating factors behind food choice as assessed by the FCQ and statements on ethical concern – for all participants and between main diet groups.

Instrument/item	Motivation domain	All participants (*n* = 444)	Omnivore group (*n* = 374)	Vegan/vegetarian group (*n* = 70)	Between-group comparisons (vegan/vegetarian vs. omnivore)
FCQ (mean ± SD)	Health	2.79 ± 0.70	2.77 ± 0.72	2.94 ± 0.59	*Z* = −1.811, *p* = 0.070
	Mood	2.35 ± 0.76	2.30 ± 0.75	2.63 ± 0.78	Z = −3.286, *p* = 0.001
	Convenience	2.91 ± 0.60	2.92 ± 0.59	2.89 ± 0.63	*Z* = −0.069, *p* = 0.945
	Sensory Appeal	2.96 ± 0.63	2.96 ± 0.64	2.99 ± 0.63	*Z* = −0.633, *p* = 0.527
	Natural Content	2.51 ± 0.89	2.43 ± 0.90	2.91 ± 0.78	*Z* = −4.193, *p* = 0.000
	Price	3.03 ± 0.71	3.05 ± 0.72	2.95 ± 0.66	*Z* = −1.158, *p* = 0.247
	Weight Control	2.36 ± 0.79	2.31 ± 0.79	2.59 ± 0.75	*Z* = −2.959, *p* = 0.003
	Familiarity	2.33 ± 0.76	2.30 ± 0.76	2.48 ­± 0.79	*Z* = −1.907, *p* = 0.056
	Ethical Concern	2.04 ± 0.82	1.95 ± 0.79	2.52 ± 0.78	*Z* = −5.447, *p* = 0.000
Additional statements on Ethical Concern (mean ± SD)	*‘…* does not involve the harm or exploitation of animals*’*	2.29 ± 1.07	2.09 ± 0.99	3.33 ± 0.85	*Z* = −8.571, *p* = 0.000
	*‘*… does not involve the killing of animals*’*	1.87 ± 1.08	1.61 ± 0.89	3.27 ± 0.92	*Z* = −10.872, *p* = 0.000
	‘… is more environmentally friendly’	2.29 ± 0.99	2.14 ± 0.94	3.11 ± 0.81	*Z* = −7.529 *p* = 0.000
	‘… is fair-trade’	2.20 ± 0.97	2.07 ± 0.93	2.90 ± 0.84	*Z* = −6.647, *p* = 0.000
	‘… is organic’	2.20 ± 1.02	2.09 ± 1.0	2.80 ± 0.91	*Z* = −5.452, *p* = 0.000

FCQ = Food Choice Questionnaire (Steptoe et al., 1995); SD = standard deviation.

**Table 6.** Single most important motivational domain from the Food Choice Questionnaire (FCQ) – plus our additional statements – as listed by all participants and between main diet groups.

Item	Motivation domain	All participants (*n* = 444)	Omnivore group (*n* = 374)	Vegan/vegetarian group (*n* = 70)
The single most important motivational domain (*n* (%) participants)	Health	156 (35.1%)	120 (32.1%)	36 (51.4%)
	Mood	21 (4.7%)	15 (4.0%)	6 (8.6%)
	Convenience	37 (8.3%)	35 (9.4%)	2 (2.9%)
	Sensory Appeal	84 (18.9%)	77 (20.6%)	7 (10.0%)
	Natural Content	17 (3.8%)	16 (4.3%)	1 (1.4%)
	Price	79 (17.8%)	77 (20.6%)	2 (2.9%)
	Weight Control	24 (5.4%)	21 (5.6%)	3 (4.3%)
	Familiarity	10 (2.3%)	10 (2.7%)	0 (0.0%)
	^a,b^Ethical Concern - for animal welfare	13 (2.9%)	1 (0.3%)	12 (17.1%)
	^a^Ethical Concern – for the environment	1 (0.2%)	1 (0.3%)	0 (0.0%)
	^a^Ethical Concern – for fair trading	1 (0.2%)	1 (0.3%)	0 (0.0%)
	^a,c^Organic produce	1 (0.2%)	0 (0.0%)	1 (1.4%)

^a^ additional statements around ethical concerns added by authors.

^b^ two additional statements combined (‘does not involve the harm or exploitation of animals’ and ‘does not involve the killing of animals’).

^c^ organic produce could be interpreted as an ethical or a health factor in food choice.

#### Food choice motivators in vegan/vegetarians vs. omnivores

Mann-Whitney *U* tests showed statistically significant differences in FCQ scores between the two main dietary patterns of vegan/vegetarian vs. omnivore in the following motivational domains: Mood *Z* = −3.286, *p* = 0.001 (vegan/vegetarian mean 2.63 ± SD 0.78 vs. omnivore mean 2.30 ± SD 0.75); Natural Content, *Z* = −4.193, *p* = 0.000 (vegan/vegetarian mean 2.91 ± SD 0.78 vs. omnivore mean 2.43 ± SD 0.90); Weight Control, *Z* = −2.959, *p* = 0.003 (vegan/vegetarian mean 2.59 ± SD 0.75 vs. omnivore mean 2.31 ± SD 0.79); and Ethical Concern, *Z* = −5.447, *p* = 0.000 (vegan/vegetarian mean 2.52 ± SD 0.78 vs. omnivore mean 1.95 ± SD 0.79). That is, the vegan/vegetarian group placed greater importance on all of these motivational domains compared with omnivores (see Table 5).

Mann-Whitney *U* tests showed statistically significant differences between vegan/vegetarian vs. omnivore groups in all five of the added questionnaire statements regarding ethical concern, all *p* = 0.000 (Table 5). Half (51.4%) of all vegan/vegetarians listed Health as the single most important motivational factor in diet choice, followed by all ethical concern additional questions combined (18.5%, Table 6). In comparison, one-third (32.1%) of omnivores listed Health as the single most important motivational factor in diet choice, followed by Sensory Appeal and Price (both 20.6%, Table 6).

#### Food choice motivations in people who score <35 on the ORTO-15 vs. those who score 35+

A Mann–Whitney *U* test showed a statistically significant difference in FCQ score in the Health domain between people who scored <35 in the ORTO-15 vs. people who scored 35+, *Z* = −2.151, *p* = 0.032 (<35 ORTO-15 mean 2.98 ± SD 0.63 vs. 35+ ORTO-15 mean 2.76 ± SD 0.71); and in the Weight Control domain between people who scored <35 in the ORTO-15 vs. people who scored 35+, *Z* = −5.082, *p* = 0.000 (<35 ORTO-15 mean 2.81 ± SD 0.66 vs. 35+ ORTO-15 mean 2.28 ± SD 0.78). That is, people who scored <35 in the ORTO-15 – indicating more orthorexic tendencies – placed greater importance on Health and Weight Control in affecting their food choice compared to those who scored 35 + on the ORTO-15. Note that a Pearson correlation between BMI and FCQ Weight Control was not significant, *r* = 0.071, *p* = 0.139. Also, Health was listed as the single most important motivational factor in food choice in all participants, regardless of scoring <35 or 35 + on the ORTO-15 (<35: *n* = 26/66, 39.4%; 35+: *n* = 130/378, 34.4%).

#### Food choice motivators in vegan/vegetarian sub-groups

A Kruskal–Wallis H test showed a statistically significant difference in Ethical Concern motivational domain scores between the seven vegan/vegetarian diet sub-patterns, χ^2^(6) = 19.726, *p* = 0.003. Lacto-vegetarians (mean 3.00 ± SD 0.99), dietary vegans (mean 2.97 ± SD 0.46), lifestyle and dietary vegans (mean 2.51 ± SD 0.57), and ovo-vegetarians (mean 2.50 ± SD 1.18) reported higher Ethical Concern scores compared to lacto-ovo-vegetarians (mean 2.12 ± SD 0.78), pescatarians (mean 2.00 ± SD 0.86), and flexitarians/semi-vegetarians (mean 2.00).

## Discussion

The aims of this online survey were to compare orthorexic, obsessively healthy tendencies between 444 male and female vegans/vegetarians and omnivores, and reasons behind food choice using the FCQ (Steptoe et al., 1995). Please see Table 3 for a summary of the main results. In support of our hypothesis, 70/444 (15.8%) of participants who reported vegan/vegetarian eating habits scored significantly lower on the ORTO-15 questionnaire (Donini et al., 2005) compared with those following an omnivorous diet, suggesting more orthorexic traits. Similarly, there were significantly more vegans/vegetarians who scored <35 on the ORTO-15, suggesting ‘orthorexia’, with an estimated prevalence of ON in this diet group of 31.4%, compared to 11.8% in the omnivores. Vegans in particular had some of the highest prevalences of ON: dietary vegans 42.9% and lifestyle and dietary vegans 30.8%. These results are in line with the finding from McLean et al.'s (2022) systematic review that vegetarianism and veganism were associated with ON overall, and with the recent 90% agreement from an international panel of experts on ON that vegan and vegetarian eating habits may be a risk factor for, or a characteristic of, ON (Donini et al., 2022); and also corroborates findings that risk of ON can be highest in vegans (58.2%, Parra-Fernández et al., 2020). Additionally, we found an association between vegan/vegetarian eating styles and past or current eating disorders, as per previous research (Bardone-Cone et al., 2012), which supports ON being aligned with eating disorder diagnoses (Cena et al., 2019).

Also as per our hypotheses, we reported a significant inverse relationship between ORTO-15 score and FCQ scores in the Health Concern domain, suggesting that choosing food for health reasons is associated with more orthorexic traits. That is, the higher the health motivators, the greater the risk of that motivation becoming unhealthy. This is to be expected given that in ON there is an obsession with consuming a healthy diet (Donini et al., 2022, Cena et al., 2019). It is also consistent with the findings from Barthels et al. (2020), who reported an association between orthorexic traits and dietary health motivations in a sample of vegan people. Additionally, our results regarding the Health Concern FCQ domain being significantly associated with the risk of ON supports the orthorexic trait-health motivation connection (people with an ORTO-15 score of <35 had average FCQ Health scores closer to 3, ‘moderately important’ (2.98), than people with an ORTO-15 score of 35+ (2.76)).

However, we did not find a significant difference between vegans/vegetarians and omnivores in the FCQ Health Concern domain. One would think that plant-centric eating patterns would be more driven by health reasons than in animal-focused diets (e.g., avoiding processed meat products to avoid non-communicable disease (WHO, 2021, Godfray et al., 2018)), yet Health Concern was listed as the single most important factor in food choice in *both* vegan/vegetarian and omnivorous populations in our study.

In contrast, there were significant differences between vegans/vegetarians and omnivores in the FCQ domains of Ethical Concern (as per our hypotheses), Weight Control, Natural Content, and Mood – with vegans/vegetarians scoring higher in all domains by around 0.5, with average FCQ scores for these domains being closer to 3 (moderately important), while omnivore scores were closer to 2 (a little important). This suggests that people in our sample who followed plant-focused diets compared with those who followed animal-focused diets were more driven by: Concern for humans, other animals, and the environment; achieving a healthy body weight; consuming more natural vs. artificial content; and eating to feel happy and cope with stress.

With regard to vegans/vegetarians scoring significantly higher than omnivores in the FCQ ethics domain, Hessler-Kaufmann et al. (2021) also reported that vegetarians had higher ecological/ethical eating motives compared to semi-vegetarians and omnivores. Similarly, Kim et al. (2022) found that vegetarians scored higher in the ethical domain of the FCQ compared to omnivores. We also found significant differences between the vegan/vegetarian vs. omnivore groups in all five of the added questionnaire statements that were regarding ethical concern for animals, the environment, and other humans (fair-trade), i.e., the vegan/vegetarian group scored higher for all statements than the omnivore group, highlighting higher ethical concern. With environmental ethics, our findings make sense because evidence suggests that animal farming and meat consumption has major negative effects on climate change (Godfray et al., 2018), and that a vegan diet is optimal for the environment due to the production of the lowest amount of greenhouse gas (Chai et al. 2019).

With regard to vegans/vegetarians scoring significantly higher than omnivores in the FCQ body weight domain, our results again are to be expected, because most evidence suggests that plant-based diets improve weight status (Tran et al., 2020). However, Parra-Fernández et al. (2020) and Kim et al. (2022) report the opposite trend in body weight between plant-based and omnivorous groups, with vegans/vegetarians being less driven by weight control motivators than omnivores in these studies. Additionally, we found that the Weight Control FCQ domain was significantly associated with scoring <35 on the ORTO-15 (with average FCQ Weight Control scores being closer to 3 (‘moderately important’, (2.81)), while the 35+ ORTO-15 group had average FCQ Weight Control scores closer to 2 (‘a little important’, (2.28)), and also significantly inversely related to ORTO-15 score, i.e., people who completed our survey who were more motivated by body weight control factors (either vegan/vegetarian or omnivorous) were more likely to have obsessive tendencies about the healthfulness and purity of their diet. Similarly, a 2022 systematic review on ON and eating disorder behaviours found that ON was consistently related to weight control motivations for food choice in the review's included research (Atchison & Zickgraf, 2022). However, Parra-Fernández et al., 2020 did not find any weight-ON correlation. ON is different from other eating disorders in that the several proposed diagnostic criteria for it specify that any desire for weight loss must be absent (Cena et al., 2019; Donini et al., 2022). It is still to be determined if body weight is a defining characteristic of ON or just one possible aspect of it.

With regard to vegans/vegetarians scoring significantly higher than omnivores in the FCQ natural content domain, Parra-Fernández et al. (2020) also reported that vegetarians and vegans were more motivated by the ‘clean label’ aspect of food than omnivores, such as organic farming methods which are more ‘natural’ because they do not involve the use of synthetic, human-made chemicals (Asioli et al., 2017).

With regard to vegans/vegetarians scoring significantly higher than omnivores in the FCQ emotion regulation domain, it makes sense that food choices in vegans and vegetarians might be more laden with feelings in general, because it takes a level of passion to change one's diet in an attempt to improve the lives of humans and other animals, and to benefit the environment. If a vegan was feeling hopeless about anything in his or her life, taking action in food choice during one meal may result in feelings of empowerment and improved mood. Additionally, heightened emotions around food choice in plant-based eating patterns may be related to our findings that vegans/vegetarians were more likely to have orthorexic traits and have a diagnosis of an eating disorder, and to previous evidence linking vegan/vegetarian diets to disordered eating (Bardone-Cone et al., 2012). However, not all studies report differences in plant- vs. animal-based eating patterns with FCQ mood (Kim et al., 2022); and Atchison's 2022 review reports that ON is less consistently related to the eating disorder symptom of emotional eating compared with other eating disorder symptoms. Exploring the links between plant-based eating, disordered eating, and other mental health symptoms and conditions (such as depression, Hessler-Kaufmann et al., 2020) is important to be able to better understand how different dietary patterns affect the human mind, and vice versa.

Strengths of our study include: Involving more male participants than most previous studies (McLean et al., 2022); and being the first study to date to examine plant-based eating patterns, ON, and diet motivations using a reliable and validated motivation-based questionnaire in English (Parra-Fernández et al., 2020 were the only other authors to use a validated FCQ, but in Spanish,). Also, the internal consistency reliability of the tools we used in the current study was moderate to strong. Lastly, MTurk has been touted as ‘an efficient, reliable, cost-effective tool for generating sample responses that are largely comparable to those collected via more conventional means’ (Mortensen & Hughes, 2018), e.g., Buhrmester et al., 2011 reported that 116 MTurk participants demonstrated similar test-retest reliability coefficients on measures of self-esteem compared with results from a traditional data collection method in undergraduate students by Bosson et al., 2000 (test–retest reliability correlation 0.87; *p* < 0.01).

Limitations of our study include: The cross-sectional nature of survey methodology, which shows correlation and not causation; the self-reported nature of diet patterns; results gained from MTurk may not be generalisable to wider populations (Mortensen & Hughes, 2018); some evidence suggesting that the different items of the FCQ may be understood differently across cultures (Cunha et al., 2018); the lack of background testing for the five additional statements that we added to the FCQ Ethical Concern dimension (although we did not include these when calculating average scores for FCQ Ethical Concern, hence not altering the original FCQ instrument scoring (Steptoe et al., 1995)) and the possible interpretation of the ‘organic’ additional item as being an environmental ethics statement *or* a health statement, e.g., non-organic produce containing pesticides/toxins/chemicals (Donini et al., 2022); and the possible psychometric limitations of the ORTO-15 and the possibility that it may not indicate pathological eating behaviours but instead normal health conscious ones in what has been called ‘healthy orthorexia’ (Depa et al., 2019).

In conclusion, we add to the evidence base in a younger US population comprising more males that vegan/vegetarian diets (15.8% of people) were associated with greater orthorexic tendencies, and vegan/vegetarians chose food more often for Weight Control, Ethical Concern, Natural Content, and Mood reasons. Additionally, people with greater orthorexic tendencies chose food significantly more often for Weight Control and Ethical Concern reasons than those with less orthorexic tendencies. Future studies would ideally use interviews to complement data collected from questionnaires, in a larger population that includes all sexes and several Western and non-Western cultures, also monitoring change over time. Any of the body weight-motivation components of ON should be explored in detail, and assessment of ON should be done using the most reliable tool available at the time. Disordered eating and food motivations should also be assessed using validated tools and/or interviews. Working out what motivates people who follow plant-based eating patterns is of particular importance in view of the need to improve the ethical aspects of the global food chain, i.e., reducing animal product consumption to help with nutrition-related global challenges such as animal rights and climate change, as well as the grand problem of diet-related disease. Additionally, how do we tailor messages in public health marketing to promote plant-based diets for human, animal, and environmental health, whilst also preventing any plant-fuelled healthy eating tendencies tipping into orthorexic obsessions?

## Supplemental Material

sj-docx-1-nah-10.1177_02601060231187924 - Supplemental material for Vegan and vegetarian males and females have higher orthorexic traits than omnivores, and are motivated in their food choice by factors including ethics and weight control Supplemental material, sj-docx-1-nah-10.1177_02601060231187924 for Vegan and vegetarian males and females have higher orthorexic traits than omnivores, and are motivated in their food choice by factors including ethics and weight control by Rebecca Reynolds, Andrea McGowan, Sophie Smith and Patrick Rawstorne in Nutrition and Health
