# Supplementary material for: Vegan and vegetarian males and females have higher orthorexic traits than omnivores, and are motivated in their food choice by factors including ethics and weight control
Source: Nutr Health. 2023 Jul 19;31(2):439–50. doi: 10.1177/02601060231187924 (PMC12174629; doi:10.1177/02601060231187924)
Supplement: sj-docx-1-nah-10.1177_02601060231187924 - Supplemental material for Vegan and vegetarian males and females have higher orthorexic traits than omnivores, and are motivated in their food choice by factors including ethics and weight control [file sj-docx-1-nah-10.1177_02601060231187924.docx]

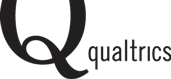


# Participant Information Statement

**Questionnaire on motivating factors behind vegan and vegetarian eating patterns**

## Please read the Participant Information Statement [here](https://drive.google.com/file/d/1enMVjEbyBz4hg2Vh0mRC2GEkfEoeFrk1/view?usp=sharing) before continuing.

...

## Please read the following and check to agree:


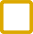
 I understand I am being asked to provide consent to participate in this research study;


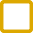
 I have read the Participant Information Statement and it has been provided to me in a language that I understand;


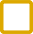
 I provide my consent for the information collected about me to be used for the purpose of this research study only;


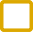
 I understand that if necessary I can ask questions and the research team will respond to my questions;


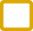
 I freely agree to participate in this research study as described and understand that I am free to withdraw at any time during the study and withdrawal will not affect my relationship with any of the named organisations and/or research team members;


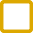
 I would like to be entered into the study’s prize draw and also receive a copy of the study results via email. I have provided my email address below and ask that it is used for these purposes only;


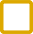
 I understand that I can download a copy of this consent form from [here](https://drive.google.com/file/d/1enMVjEbyBz4hg2Vh0mRC2GEkfEoeFrk1/view?usp=sharing).

Email address so that I can be entered in to the prize draw and be informed of the results of the study

If you have provided your email address and at any time you wish to withdraw your consent to participate in the study, please complete [this form](https://drive.google.com/open?id=1_oKHHQ_QuwywRIneA3gz_WXUM-a7txdN). You are not able to withdraw your consent if you have not provided your email address.

# Screening Criteria

## Are you 16 years or older?


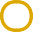
 Yes
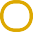
 No

## Is your first language (mother tongue) English?


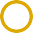
 Yes
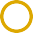
 No

You are not eligible to complete the survey.

This is because you do not meet our age requirements or your first language (mother tongue) is not English.

If you have any questions, please email [rebecca.reynolds@unsw.edu.au](mailto:rebecca.reynolds@unsw.edu.au)

# Demographics

## How old are you?

What gender do you identify with?


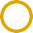
 Male
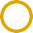
 Female
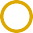
 Other

## Where do you live?


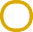
 United States


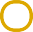
 United Kingdom (England, Northern Ireland, Scotland, and Wales)
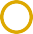
 Australia


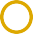
 New Zealand


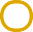
 Other (please specify)

## If you live in Australia, which state or territory do you live in?


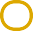
 Australia Capital Territory

New South Wales
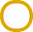
 Victoria


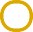
 Queensland
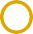
 South Australia


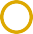
 Western Australia
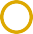
 Tasmania


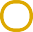
 Northern Territory

## What is your highest level of education completed so far?


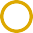
 Below year 12 (did not finish high school)
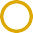
 Year 12 (finished high school)


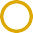
 Diploma or similar


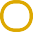
 Bachelor degree or above

## What race or ethnicity do you best identify with?


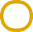
 Oceanian: Australian


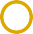
 Oceanian: Australian Aboriginal


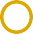
 Oceanian: Australian South Sea Islander
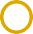
 Oceanian: Australian Torres Strait Islander
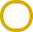
 Oceanian: Other


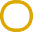
 North-West European


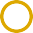
 Southern and Eastern European
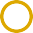
 North African and Middle Eastern
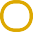
 South-East Asian


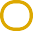
 North-East Asian


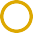
Southern and Central Asian

People of the Americas
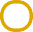
 Sub-Saharan Africa


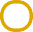
 Other


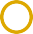
 Prefer not to specify

## What best describes your dietary patterns?


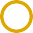
 Omnivore (consume animal foods and drinks as well as plant foods and drinks)
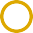
 Vegan or vegetarian (avoid most or all animal products)

# Medical History

What is your weight in kg? (Please convert pounds to kg, e.g. using [this](https://www.google.com.au/search?q=convert%2Bpounds%2Bto%2Bkg&oq=convert%2Bpounds%2Bto&aqs=chrome.0.0j69i60j69i57j0l3.2459j0j4&sourceid=chrome&ie=UTF-8) Google online calculator)

What is your height in cm? (Please convert inches to cm, e.g. using [this](https://www.google.com.au/search?ei=CYqgWpOnFMX10gTdwp_ACQ&q=convert%2Binches%2Bto%2Bcm&oq=convert%2Binches%2Bto%2Bcm&gs_l=psy-ab.3..0l2j0i20i263k1j0l7.7152.9593.0.9817.20.13.3.0.0.0.344.1982.0j5j3j1.9.0....0...1c.1.64.psy-ab..9.11.1822...0i67k1j0i10k1.0.rz3ddHRA1BI) Google online calculator)

Have you ever been clinically diagnosed with any of the following eating disorders, i.e. in the past or now? If so specify which one/s.


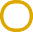
 No, I have not been clinically diagnosed with an eating disorder
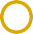
 Anorexia nervosa


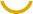
Bulimia nervosa


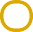
 Binge eating disorder
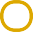
 Pica


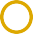
 Rumination disorder


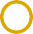
 Avoidant/restrictive food intake disorder
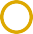
 Other specified feeding or eating disorder
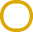
 Unspecified feeding or eating disorder

## Do you have any micronutrient deficiencies? If so please specify what deficiency/deficiencies and if you take anything to correct it, e.g. iron supplement.


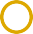
 Yes
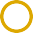
 No

## Do you take any supplements? If so, please specify what you take.

Have you recently lost weight?


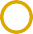
 No


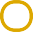
 Unsure


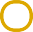
 Yes (1 - 3 kg)


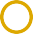
 Yes (>3 - 6 kg)


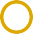
 Yes (>6 - 9 kg)
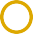
 Yes ( > 9 kg)

## Please check any of the following illnesses that you have been clinically diagnosed with. If so, please specify who diagnosed the illness, e.g. GP.


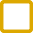
 Crohn's disease
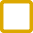
 Coeliac disease
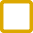
 Diverticulitis


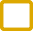
 Irritable bowel syndrome
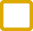
 Ulcerative colitis


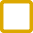
 Diabetes


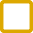
 Chronic constipation
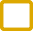
 Chronic diarrhoea


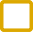
 Any other medical condition you wish to report
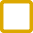
 None of the above

## Do you have any food intolerances or allergies? If so, please specify who diagnosed the allergy, e.g. a specialist doctor, such as an immunologist; or you used an allergy testing service that tested for IgG antibodies; etc.


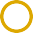
 Yes
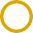
 No

# ORTO 15

## Answer the following questions to the best of your ability.

Always Often Sometimes Never


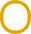

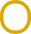

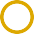

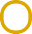

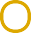

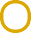

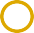

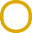


When eating, do you pay attention to the calories of the food?

When you go in a food shop do you feel confused?


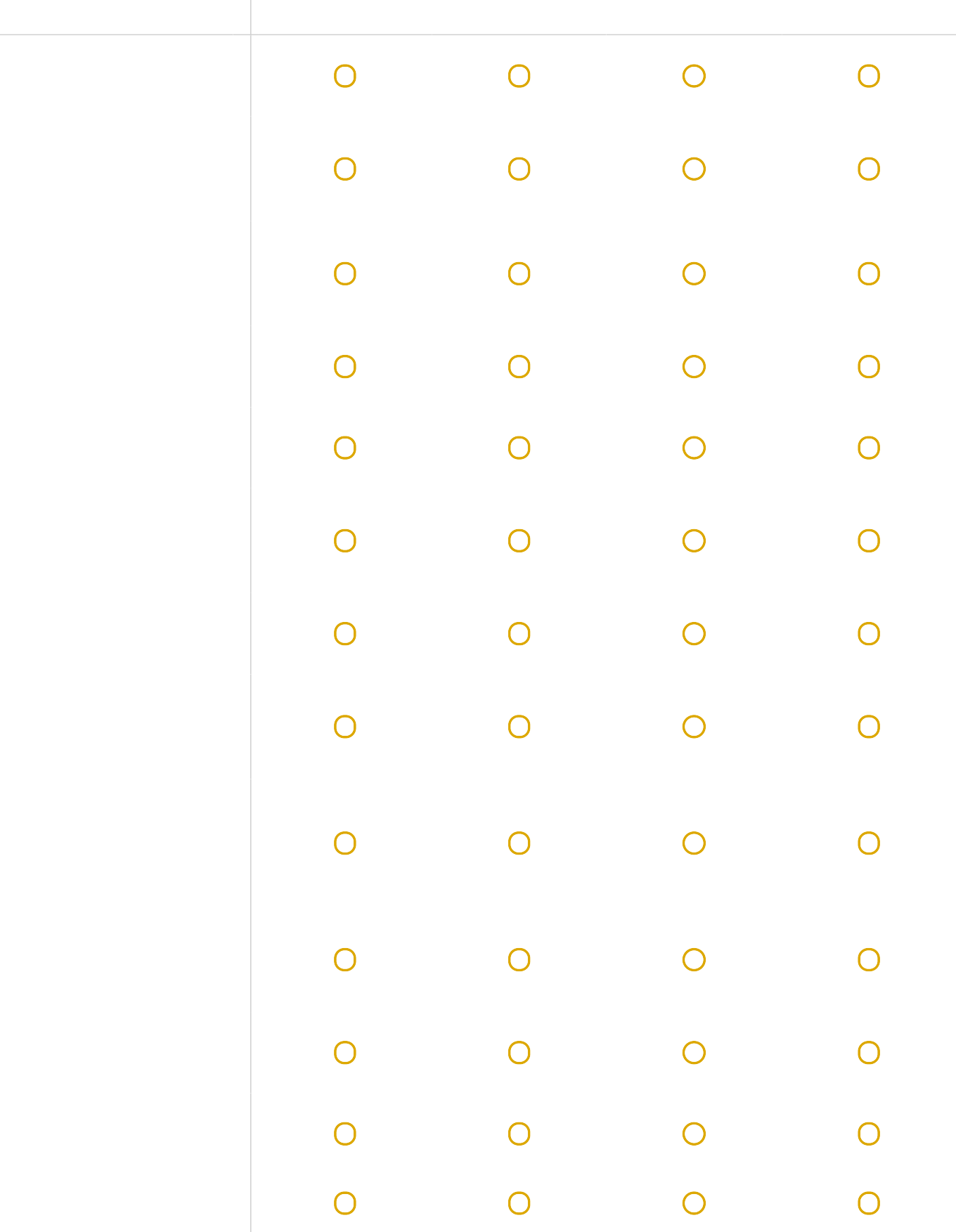
Always Often Sometimes Never

In the last 3 months, did the thought of food worry you?

Are your eating choices conditioned by your worry about your health status?

Is the taste of food more important than the quality when you evaluate food?

Are you willing to spend more money to have healthier food?

Does the thought about food worry you for more than three hours a day?

Do you allow your self any eating transgressions ('slip- ups')?

Do you think your mood affects your eating behavior?

Do you think that the conviction (decision) to eat only healthy food increases self-esteem?

Do you think that eating healthy food changes your lifestyle (frequency of eating, friends...etc)

?

Do you think that consuming healthy food may improve your appearance?

Do you feel guilty when transgressing ('slipping up' with your diet)?

Do you think that on the market there is also unhealthy food?

Are you (usually) alone when having meals?

# Food Choice Questionnaire

## It is important to me that the food I eat on a typical day.......


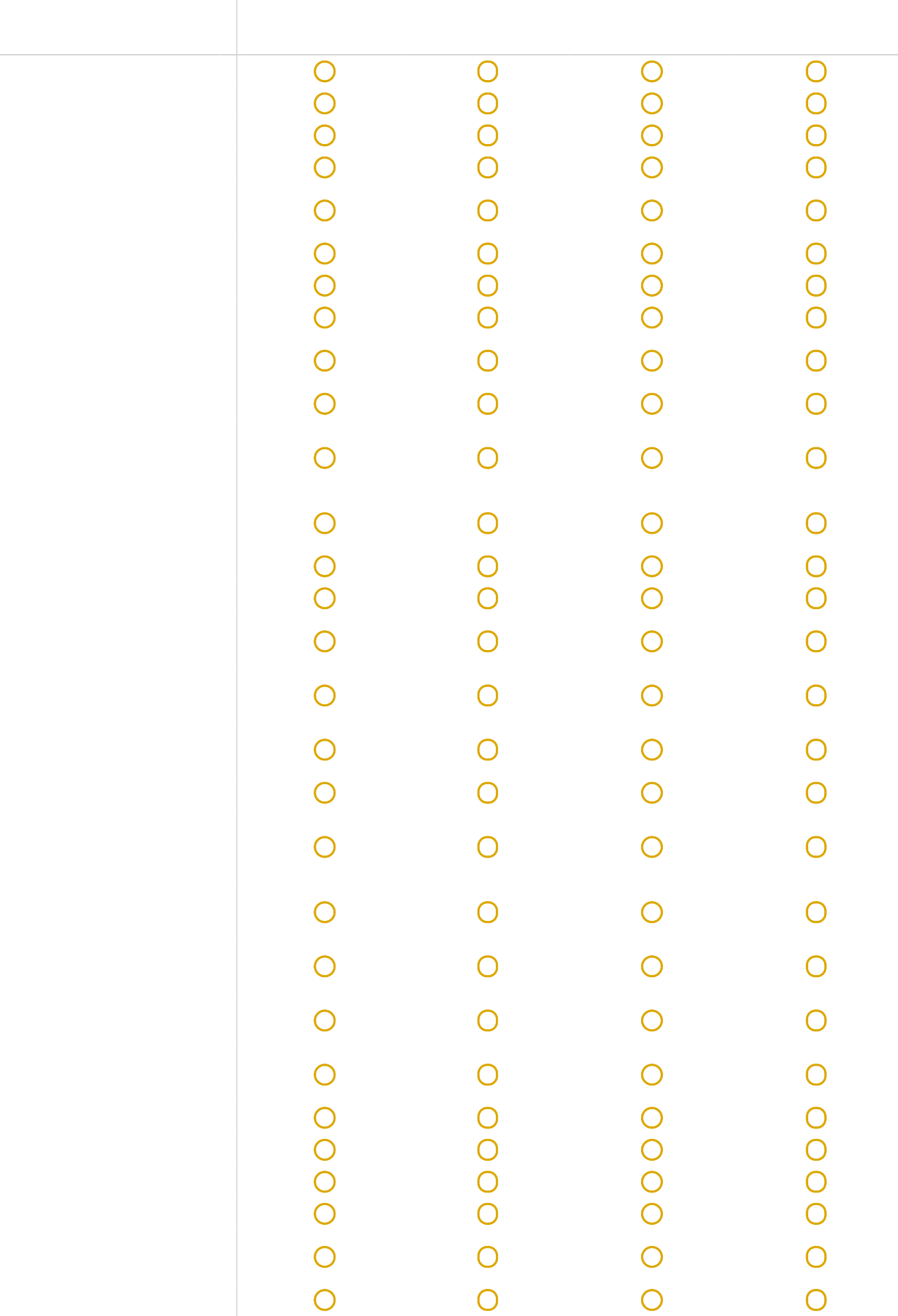


...is easy to prepare

...contains no additives

...is low in calories

...tastes good

...contains natural ingredients

...is not expensive

...is low in fat

...is familiar

...is high in fibre and roughage

...is nutritious

...is easily available in shops and supermarkets

...is good value for money

...cheers me up

...smells nice

...can be cooked very simply

...helps me cope with stress

...helps me control my weight

...has a pleasant texture

...is packaged in an environmentally friendly way

...comes from countries I approve of politically

...is like the food I ate when I was a child

...contains a lot of vitamins and minerals

...contains no artificial ingredients

...keeps me awake/alert

...looks nice

...helps me relax

...is high in protein

...takes no time to prepare

...keeps me healthy

Not at all

important A little important

Moderately

important Very important

...is good for my skin/teeth/hair/nails etc

...makes me feel good

...has the country of origin clearly marked

...is what I usually eat

...helps me to cope with life

...can be bought in shops close to where I live or work

...is cheap

...does not involve the harm or exploitation of animals

...does not involve the killing of animals

...is more environmentally friendly

...is fair-trade

...is organic

Not at all


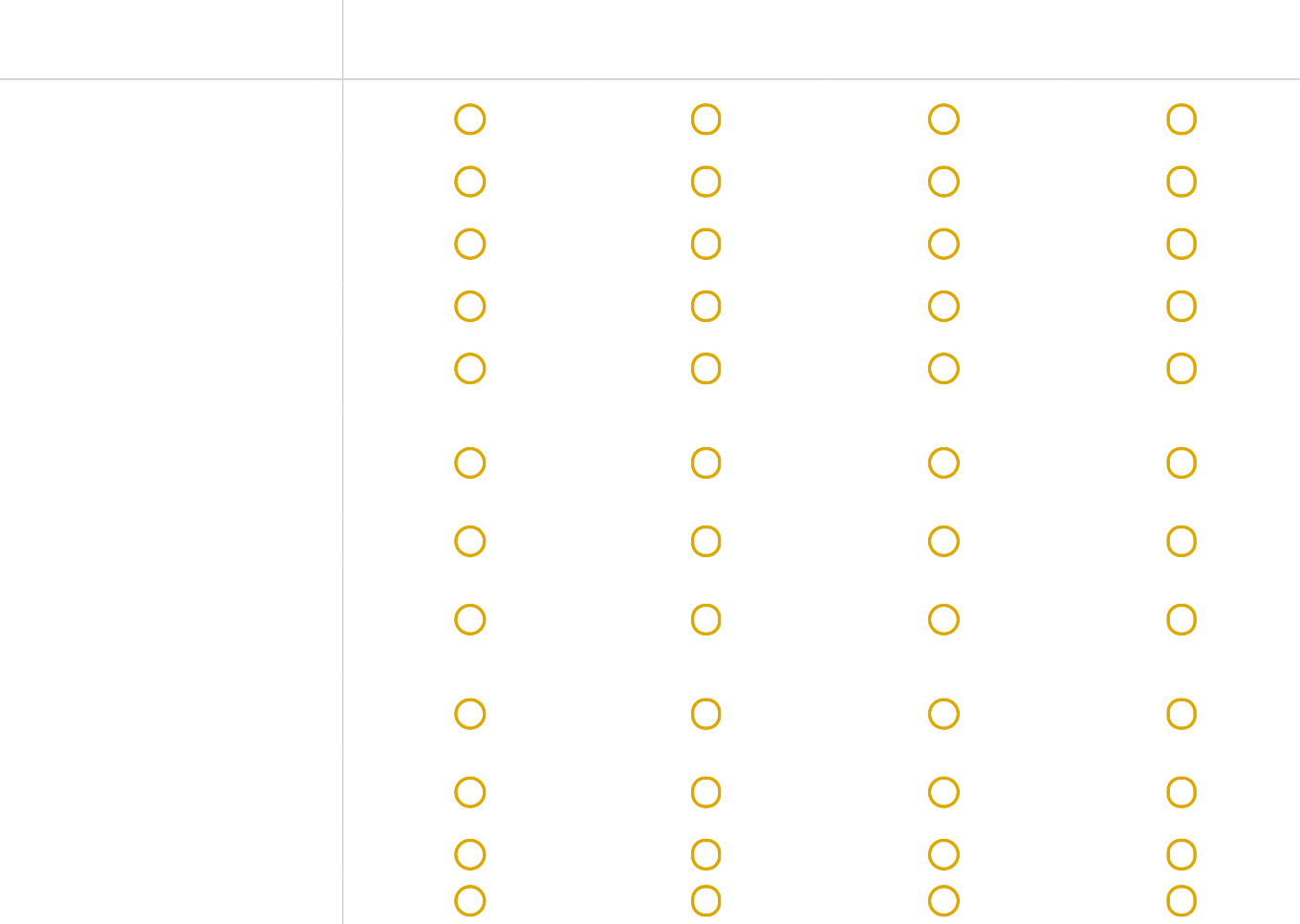
important A little important

Moderately

important Very important

## From all of the options above, select the most important factor for you in food selection.


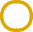
 Health (e.g. good for physical health like skin)
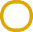
 Mood (e.g. makes me feel good)


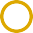
 Convenience (e.g. easy to prepare)
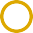
 Sensory appeal (e.g. tastes good)


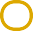
 Natural content (e.g. contains no additives)
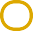
 Price (e.g. good value)


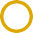
 Weight control (e.g. helps me control my weight)
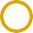
 Familiarity (e.g. what I am used to eating)


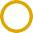
 Ethical concern for animal welfare (e.g. doesn't harm or exploit animals)
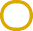
 Ethical concern for environment


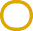
 Ethical concern for fair trading


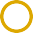
 Organic produce (please specify why you choose organic produce)

# Eating Habits - Omnivore

## What best describes your current dietary pattern?


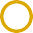
 Animal-heavy omnivore: relying more on animal foods/drinks than plant foods/drinks for nutrition (i.e. consume animal foods or drinks 5 or more times per day)

Animal-moderate omnivore:relying equally on animal and plant foods/drinks for nutrition (i.e. consume animal foods or drinks 3-4 times per day)

Plant-heavy omnivore: relying more on plant foods/drinks than animal foods/drinks for nutrition ( i.e. consume animal foods or drinks 2 or less times per day)

## Do you ever try to reduce your animal food/drink consumption (e.g. milk, chicken, beef, pork, eggs, cheese, fish, turkey etc)?

Frequently Sometimes Never

## Have you every considered becoming a vegetarian or vegan?

Yes No

I have been one in the past

## Aside from being omnivorous (consuming both plants and animals), do you follow any of the following dietary patterns? If so please explain why.

Paleo Raw diet

Gluten-free Ketogenic diet Other

None of the above

# Why limit meat motivation

## If you limit your meat consumption, what is the motivation behind that decision?

Health: it is better for my physical health to consume fewer animal products

Environmental: it is less taxing on the environment to consume fewer animal products

Ethical: it is better for animal welfare to consume fewer animal products Cost: it is more expensive to consume animal products

Convenience: it is more convenient to consume fewer animal products I never limit my meat consumption

# Thank you

Thank you for your participation. If you have provided an email address, expect to hear study results and fingers crossed that you win one of the $50 prizes available. If you have any questions or concerns, please contact [rebecca.reynolds@unsw.edu.au](mailto:rebecca.reynolds@unsw.edu.au)

If you have provided your email address, but no longer wish to take part in the study, please complete [this form](https://drive.google.com/open?id=1_oKHHQ_QuwywRIneA3gz_WXUM-a7txdN).

# Eating Habits- VV

## Current dietary classification? Select which applies best.

Dietary vegan: never consume meat, dairy, eggs; but use non-vegan clothing and beauty products

Lifestyle and dietary vegan - never consume meat, dairy, eggs, honey; and avoid usage of beauty products and clothing that exploit or harm animals

Lacto-ovo-vegetarian: never consume meat, fish or fowl (e.g. chicken); but consume eggs and dairy

Pescatarian: never consume meat or fowl (e.g. chicken); but consume fish, eggs and dairy

Lacto-vegetarian:- never consume eggs, meat, fish or fowl (e.g. chicken); but consume dairy

Ovo-vegetarian- never consume dairy, meat, fish or fowl (e.g. chicken); but consume eggs

Flexitarian/semi-vegetarian: consume meat, fish or fowl (e.g. chicken) more than 1 time per month, but less than 1 time per week; and eggs/dairy at any frequency

## How long have you followed your current dietary pattern?

< 1 month

< 6 months

6 - 12 months

1 - 2 years

2 + years Entire life

## Have you ever followed any of the following dietary patterns?

I have tried this before

Dietary vegan: never consume meat, dairy, eggs; but use non- vegan clothing and beauty products

Lifestyle and dietary vegan - never consume meat, dairy, eggs, honey; and avoid usage of beauty products and clothing that exploit or harm animals

Lacto-ovo-vegetarian: never consume meat, fish or fowl (e.g. chicken); but consume eggs and dairy

Pescatarian: never consume meat or fowl (e.g. chicken); but consume fish, eggs and dairy

Lacto-vegetarian:- never consume eggs, meat, fish or fowl (e.g. chicken); but consume dairy

Ovo-vegetarian- never consume dairy, meat, fish or fowl (e.g. chicken); but consume eggs

Flexitarian/semi- vegetarian: consume meat, fish or fowl (e.g. chicken) more than 1 time per month, but less than 1 time per week; and eggs/dairy at any frequency

Omnivore - consume both plant and animal foods/drinks at varying frequencies

Other (please specify)

## What dietary pattern did you follow immediately before your current one?

Dietary vegan: never consume meat, dairy, eggs; but use non-vegan clothing and beauty products

Lifestyle and dietary vegan - never consume meat, dairy, eggs, honey; and avoid usage of beauty products and clothing that exploit or harm animals

Lacto-ovo-vegetarian: never consume meat, fish or fowl (e.g. chicken); but consume eggs and dairy

Pescatarian: never consume meat or fowl (e.g. chicken); but consume fish, eggs and dairy

Lacto-vegetarian:- never consume eggs, meat, fish or fowl (e.g. chicken); but consume dairy

Ovo-vegetarian- never consume dairy, meat, fish or fowl (e.g. chicken); but consume eggs

Flexitarian/semi-vegetarian: consume meat, fish or fowl (e.g. chicken) more than 1 time per month, but less than 1 time per week; and eggs/dairy at any frequency

Omnivore - consume both plant and animal foods/drinks at varying frequencies Other

[Powered by Qualtrics](http://www.qualtrics.com/)
